# Atg4 plays an important role in efficient expansion of autophagic isolation membranes by cleaving lipidated Atg8 in *Saccharomyces cerevisiae*

**DOI:** 10.1371/journal.pone.0181047

**Published:** 2017-07-13

**Authors:** Eri Hirata, Yoshikazu Ohya, Kuninori Suzuki

**Affiliations:** 1 Department of Integrated Biosciences, Graduate School of Frontier Sciences, The University of Tokyo, Kashiwa, Chiba, Japan; 2 Bioimaging Center, Graduate School of Frontier Sciences, The University of Tokyo, Kashiwa, Chiba, Japan; Niigata Daigaku, JAPAN

## Abstract

Autophagy, an intracellular degradation system, is highly conserved among eukaryotes from yeast to mammalian cells. In the yeast *Saccharomyces cerevisiae*, most Atg (autophagy-related) proteins, which are essential for autophagosome formation, are recruited to a restricted region close to the vacuole, termed the vacuole-isolation membrane contact site (VICS), upon induction of autophagy. Subsequently, the isolation membrane (IM) expands and sequesters cytoplasmic materials to become a closed autophagosome. In *S*. *cerevisiae*, the ubiquitin-like protein Atg8 is C-terminally conjugated to the phospholipid phosphatidylethanolamine (PE) to generate Atg8-PE. During autophagosome formation, Atg8-PE is cleaved by Atg4 to release delipidated Atg8 (Atg8^G116^) and PE. Although delipidation of Atg8-PE is important for autophagosome formation, it remains controversial whether the delipidation reaction is required for targeting of Atg8 to the VICS or for subsequent IM expansion. We used an IM visualization technique to clearly demonstrate that delipidation of Atg8-PE is dispensable for targeting of Atg8 to the VICS, but required for IM expansion. Moreover, by overexpressing Atg8^G116^, we showed that the delipidation reaction of Atg8-PE by Atg4 plays an important role in efficient expansion of the IM other than supplying unlipidated Atg8^G116^. Finally, we suggested the existence of biological membranes at the Atg8-labeled structures in Atg8-PE delipidation-defective cells, but not at those in *atg2*Δ cells. Taken together, it is likely that Atg2 is involved in localization of biological membranes to the VICS, where Atg4 is responsible for IM expansion.

## Introduction

Eukaryotic cells have an intracellular degradation system, macroautophagy (hereafter autophagy), which is conserved from yeast to mammals [1]. When autophagy is induced by starvation or other stresses, most Atg proteins are recruited to a dot close to the vacuole, termed the vacuole-isolation membrane contact site (VICS) [2]. Next, the isolation membrane (IM), a membrane sac, expands by engulfing cytoplasmic materials and subsequently closes to form the autophagosome, a double-membrane organelle [3, 4]. The outer membrane of a completed autophagosome then fuses with the vacuole (lysosome in mammals) to release the autophagic body and its inner membrane compartment for degradation [3]. Currently, 41 Atg (autophagy-related) proteins have been identified in *Saccharomyces cerevisiae*, of which 19 are essential for autophagosome formation.

Among the Atg proteins involved in autophagosome formation, Atg8, a ubiquitin-like protein, is a reliable marker for monitoring progression of autophagy because it localizes to the VICS, IM, and autophagosome (hereafter, autophagy-related structures) and is ultimately transported into the vacuole [5–7]. Initially, Atg8 is synthesized with an arginine (residue 117) at the C-terminus, but is processed by the cysteine protease Atg4 to produce glycine-exposed Atg8 (Atg8^G116^) [8]. Subsequently, Atg8 undergoes a ubiquitin-like conjugation reaction involving Atg7 and Atg3, an E1- and an E2-like enzyme, respectively; the Atg16·Atg5-Atg12 complex serves as an E3-like enzyme in this reaction. Finally, Atg8 is conjugated to the phospholipid phosphatidylethanolamine (PE), which anchors it to membranes [9]. Atg4 also enzymatically cleaves the amide bond between Atg8 and PE to facilitate their recycling and promote autophagosome formation [5, 8].

Cleavage of the arginine residue of Atg8 by Atg4 (hereafter, ‘cleavage’) is required for Atg8-PE production, which is in turn necessary for targeting of Atg8 to autophagy-related structures. The requirement for Atg8 cleavage can be bypassed by cells expressing Atg8^G116^ instead of full-length Atg8. Atg8^G116^ cells can produce Atg8-PE in the absence of Atg4 cleavage activity, but autophagy is still defective [5, 10, 11]. Thus, cleavage of Atg8-PE by Atg4 (hereafter, ‘delipidation’) is also important for autophagy. However, it remains unknown whether the defect in delipidation affects targeting of Atg8 to the VICS or subsequent IM expansion.

In this study, we bypassed the cleavage reaction using Atg8^G116^ cells and visualized the IM as a cup-shaped structure under the fluorescence microscope by overexpressing precursor Ape1, a selective cargo of autophagosomes [2]. This analysis showed that the delipidation reaction did not affect Atg8 targeting to the VICS and that IM expansion was severely impaired in delipidation mutants. We conclude that delipidation of Atg8-PE is important for efficient IM expansion.

## Materials and methods

### Plasmids

The plasmids and primers used in this study are listed in Tables 1 and 2. Plasmid pRS314[mNeonGreen-Atg8] was created by replacing the GFP sequence of pRS314[GFP-Atg8] [12] with the *Bam*HI cassette of mNeonGreen, amplified from pFA6a-2×mNeonGreen-kanMX using primers mNeonGreen_F and mNeonGreen_R [13]. Next, pRS314[mNeonGreen-Atg8] was digested with *Xho*I and *Sac*I, and the fragment was cloned into pRS305 after digestion with *Xho*I and *Sac*I to generate pRS305[mNeonGreen-Atg8]. Plasmid pRS303[mNeonGreen-Atg8] was generated by cloning the mNeonGreen-Atg8 fragment from pRS314[mNeonGreen-Atg8] digested with *Xho*I and *Sac*I into pRS303 digested with the same enzymes. Plasmid pRS303[mNeonGreen-Atg8^G116^] was generated by PCR-based site-directed mutagenesis using pRS303[mNeonGreen-Atg8] as a template and Atg8deltaRF and Atg8deltaRR as primers. pRS316[Atg4] was digested with *Xho*I and *Sac*I, and the fragment was cloned into pRS314 after digestion with the same enzymes to generate pRS314[Atg4].

**Table 1:** Plasmids used in this study.

Name	Alias	Properties	Marker	Source
pYEX-BX[prApe1]		Plasmid for expression of the Ape1 proform from the *CUP*1 promoter	*URA3*	[2]
pRS424[prApe1]		Plasmid for expression of the Ape1 proform from the *CUP*1 promoter	*TRP1*	[2]
pRS314[GFP-Atg8]		Plasmid for expression of GFP-Atg8	*TRP1*	[12]
pRS314		Centromeric plasmid	*TRP1*	[25]
pRS424		2μ plasmid	*TRP1*	[25]
pRS426		2μ plasmid	*URA3*	[25]
pRS316		Centromeric plasmid	*URA3*	[25]
pRS303		Integration plasmid	*HIS3*	[25]
pRS305		Integration plasmid	*LEU2*	[25]
pFA6a-hphNT1		Plasmid for gene disruption	*hphNT1*	[26]
pYEX-BX		2μ plasmid	*URA3*	Clontech
pRS306[Atg8^G116^]Δ*Cla*I		plasmid for integration of Atg8^G116^	*URA3*	Lab stock
pRS316[Atg4]	pNAK274	Plasmid for expression of Atg4	*URA3*	Provided by Dr. Hitoshi Nakatogawa
pRS426[Atg8]		Plasmid for overexpression of Atg8	*URA3*	Provided by Dr. Hitoshi Nakatogawa
pRS426[Atg8^G116^]		Plasmid for overexpression of Atg8^G116^	*URA3*	Provided by Dr. Hitoshi Nakatogawa
pRS314[GFP-Atg8^G116^]		Plasmid for expression of GFP-Atg8^G116^	*URA3*	Provided by Dr. Yoshinori Ohsumi
pFA6a-2×mNeonGreen-kanMX		Plasmid for C-terminal integration of 2×mNeonGreen	*KanMX*	Provided by Dr. Yoshinori Ohsumi
pRS314[Atg4]		Plasmid for expression of Atg4	*TRP1*	This study
pRS314[mNeonGreen-Atg8]	pYO3283	Plasmid for expression of mNeonGreen-Atg8	*TRP1*	This study
pRS305[mNeonGreen-Atg8]	pYO3284	Plasmid for integration of mNeonGreen-Atg8	*LEU2*	This study
pRS303[mNeonGreen-Atg8]		Plasmid for integration of mNeonGreen-Atg8	*HIS3*	This study
pRS303[mNeonGreen-Atg8^G116^]	pYO3287	Plasmid for integration of mNeonGreen-Atg8^G116^	*HIS3*	This study

**Table 2:** Primers used in this study.

Name	Sequence
APG04N-500F	GCCCTTCCTGCTTGTAGGTCAG
APG04C+600R	CCCACCTCTATTCATCAAATCTTCAC
ATG11N-800F	CATCATCGAGTGTTTTTCCTTTTATGTGGCC
ATG11C+500R	CCGGGTGTCGGTC
APG17N-600F	CAACCACCTCATCCTCAGAGCTC
APG17C+600R	CGTTGCATGCAGAACTACTACCATC
Atg8deltaRF	GTCACTTACTCAGGAGAAAATACATTTGGCTAGTCTTTTATATGAAAAGAAATGAAGCG
Atg8deltaRR	CGCTTCATTTCTTTTCATATAAAAGACTAGCCAAATGTATTTTCTCCTGAGTAAGTGAC
Atg2_del_Univ_F	GCATAAAGATTAAAGCAAATTAAGAGGAACCCTTTTTTTTTTTGATTTCGATACAATGCGTACGCTGCAGGTCGAC
Atg2_del_Univ_R	CGGCCGAATAATTGCCACAGGTGCAGCTCTAGCAACATAAACTGCTGCGGCGCTCGGCCCATCGATGAATTCGAGCTCG
mNeonGreen_F	AAAGGATCCGTTTCGAAAGGCGAAGAAGACAATG
mNeonGreen_R	AAAGGATCCCTTGTATAACTCGTCAG
Atg1_C_Univ_F	GATAGTATTGCAAACAGGTTGAAAATATTGAGGCAGAAGATGAACCACCAAAATGGTGGTGCAGCAGGAGGATCG
Atg1_C_Univ_R	CTTGAAAATATAGCAGGTCATTTGTACTTAATAAGAAAACCATATTATGCATCACTTAATCGATGAATTCGAGCTCG
Atg16_C_Univ_F	GGCTAAAAAAGACAGAGAAAGAGACAGAAGCCATGAACAGCGAAATAGATGGAACGAAAGGTGGTGCAGCAGGAGGATCG
Atg16_C_Univ_R	CCACAATGATTTTATTTTCTTTTGTATGCATTTTGTGACGATTTGACAACTGATGCATTAATCGATGAATTCGAGCTC

### Strains, media, and growth conditions

The yeast strains used in this study are listed in Table 3. Cells were cultured in YPD (1% Bacto^™^ yeast extract, 2% Bacto^™^ peptone, 2% glucose), SDCA (0.17% Difco^™^ yeast nitrogen base w/o amino acids and ammonium sulfate, 0.5% ammonium sulfate, 0.5% Bacto^™^ casamino acids, 2% glucose), or SDDO medium (0.17% Difco^™^ yeast nitrogen base w/o amino acids and ammonium sulfate, 0.5% ammonium sulfate, 2% glucose, appropriate nutrients for plasmid selection). Amplification of plasmids was carried out utilizing *E*. *coli* cells grown in LB medium (1% Bacto^™^ tryptone, 0.5% Bacto^™^ yeast extract, 1% NaCl). When relevant, ampicillin was added to the LB medium at a concentration of 60 μg/ml. To drive the Cu^2+^-inducible *CUP1* promoter, cells were cultured for 1 day in medium containing 250 μM CuSO_4_ prior to experiments. Autophagy was induced by addition of 0.2 μg/ml rapamycin.

**Table 3:** Yeast strains used in this study.

Strain	Genotype	Source
YCK445	SEY6210; *dpm1*Δ::*DPM1-YEGFP*:*kanMX*	[2]
KVY13	SEY6210; *atg4*Δ::*LEU2*	[8]
KVY53	SEY6210; *atg4*Δ::*LEU2 pho8*Δ::*pho8*Δ*60*	[8]
ORY0804	SEY6210; *atg8*Δ::*GFP-ATG8 atg4*Δ::*spHIS5*	[17]
SEY6210	*MATα lys2 suc2 his3 leu2 trp1 ura3*	[27]
KVY55	SEY6210; *pho8*Δ::*pho8*Δ*60*	[28]
GYS608	SEY6210; *atg8*Δ::*ATG8*^*G116*^ *atg4*Δ::*LEU2*	Lab stock
GYS622	SEY6210; *atg8*Δ::*GFP-ATG8*^*G116*^ *atg4*Δ::*LEU2*	Lab stock
GYS891	SEY6210; *ypt7*Δ::*natNT2 atg11*Δ::*cgHIS3 atg17*Δ::*hphNT1*	Lab stock
YOC5308	SEY6210; *pho8*Δ::*pho8*Δ*60 ATG8*::*ATG8*^*G116*^	This study
YOC5309	SEY6210; *atg4*Δ::*LEU2 pho8*Δ::*pho8*Δ*60 ATG8*::*ATG8*^*G116*^	This study
YOC5270	SEY6210; *atg8*Δ::*GFP-ATG8*^*G116*^ *atg4*Δ::*LEU2 atg11*Δ::*cgHIS3*	This study
YOC5269	SEY6210; *atg8*Δ::*GFP-ATG8*^*G116*^ *atg4*Δ::*LEU2 atg17*Δ::*hphNT1*	This study
YOC5271	SEY6210; *atg8*Δ::*GFP-ATG8*^*G116*^ *atg4*Δ::*LEU2 atg11*Δ::*cgHIS3 atg17*Δ::*hphNT1*	This study
YOC5209	SEY6210; *leu2*Δ::*mNeonGreen-ATG8*:*LEU2*	This study
YOC5272	SEY6210; *leu2*Δ::*mNeonGreen-ATG8*:*LEU2 atg4*Δ::*spHIS5*	This study
YOC5330	SEY6210; *atg8*Δ::*ATG8*^*G116*^ *his3*Δ::*mNeonGreen-ATG8*^*G116*^:*HIS3 atg4*Δ::*LEU2*	This study
YOC5331	SEY6210; *atg8*Δ::*ATG8*^*G116*^ *his3*Δ::*mNeonGreen-ATG8*^*G116*^:*HIS3 atg4*Δ::*LEU2 atg2*Δ::*hphNT1*	This study
YOC5469	SEY6210; *atg8*Δ::*ATG8*^*G116*^ *atg4*Δ::*LEU2 atg1*Δ::*ATG1-2×mNeonGreen-kanMX*	This study
YOC5470	SEY6210; *atg8*Δ::*ATG8*^*G116*^ *atg4*Δ::*LEU2 atg16*Δ::*ATG16-2×mNeonGreen-kanMX*	This study

To construct pho8Δ60 Atg8^G116^ (YOC5308) and pho8Δ60 Atg8^G116^ *atg4*Δ (YOC5309) strains, the pRS306[Atg8^G116^]Δ*Cla*I plasmid was digested with *Eco*RI and integrated into pho8Δ60 (KVY55) and pho8Δ60 *atg4*Δ (KVY53) strains, respectively. Then, colonies selected by SDCA (-*URA*) plates were transferred onto 5-Fluoroorotic acid (5-FOA) plates (0.17% Difco^™^ yeast nitrogen base w/o amino acids and ammonium sulfate, 0.5% ammonium sulfate, 0.5% Bacto^™^ casamino acids, 2% glucose, 0.1% 5-FOA) for gene replacement by homologous recombination.

The mNeonGreen-Atg8–expressing wild-type strain (YOC5209) was constructed by integrating pRS305[mNeonGreen-Atg8] digested with *Afl*II into the *LEU2* locus of the wild-type strain SEY6210. The mNeonGreen-Atg8^G116^ expressing Atg8^G116^ *atg4*Δ strain (YOC5330) was constructed by integrating pRS303[mNeonGreen-Atg8^G116^] digested with *Nhe*I into the *HIS3* locus of the Atg8^G116^ *atg4*Δ strain GYS608.

Gene disruptions of *ATG2*, *ATG4*, *ATG11*, and *ATG17* were performed by homologous recombination. For disruption of *ATG2*, the DNA fragment was PCR-amplified using plasmid pFA6a-hphNT1 as template and Atg2_del_Univ_F and Atg2_del_Univ_R as primers. For *ATG4* disruption, genomic DNA obtained from ORY0804 was used as a template, and APG04N-500F and APG04C+600R were used as primers. For disruption of *ATG11* and *ATG17*, genomic DNA obtained from GYS891 was used as a template and ATG11N-800F/ATG11C+500R and APG17N-600F/APG17C+600R as primers, respectively.

The Atg8^G116^ *atg4*Δ Atg1-2×mNeonGreen (YOC5469) and Atg8^G116^ *atg4*Δ Atg16-2×mNeonGreen (YOC5470) strains were constructed by transformation of the DNA fragments amplified from the pFA6a-2×mNeonGreen-kanMX plasmid into Atg8^G116^ *atg4*Δ cells using Atg1_C_Univ_F/Atg1_C_Univ_R or Atg16_C_Univ_F/Atg16_C_Univ_R as primers, respectively.

### Alkaline phosphatase (ALP) assay

The ALP assay was performed as described previously [14]. Cells were grown to mid-log phase and shifted to SD(-N) medium (0.17% Difco^™^ yeast nitrogen base w/o amino acids and ammonium sulfate, 2% glucose) to induce autophagy. Lysates were prepared by disrupting the cells with glass beads in the ice-cold ALP assay buffer (250 mM Tris-HCl (pH9.0), 10 mM MgSO_4_, 10 μM ZnSO_4_, 1 mM phenylmethylsulfonyl fluoride) and the cell debris was removed by centrifugation at 2,000 × *g* for 5 min. ALP activity in the lysate was assayed with 5.5 mM 1-naphthyl phosphate as a substrate for 10 min at 30°C, and the reaction was stopped by addition of the ALP stop buffer (2 M glycine-NaOH (pH 11.0)). The fluorescence intensity (excitation: 360nm, emission: 465nm) was measured using an RF-5300PC spectrofluorophotometer (Shimadzu).

### Sodium dodecyl sulfate–polyacrylamide gel electrophoresis (SDS-PAGE) and western blotting analysis

Cells cultured in SDCA medium (2×10^7^ cells/ml) were subjected to alkaline trichloroacetic acid (TCA) lysis followed by SDS-PAGE and western blotting analysis [15]. For analysis of Ape1 maturation, SDS-PAGE was performed using 10% acrylamide gel. For analysis of Atg8-PE formation, a 13.5% acrylamide gel containing 6 M urea was used. For analysis of GFP-Atg8 cleavage, 12% acrylamide gel was used. Proteins were transferred to polyvinylidene fluoride (PVDF) membranes (Immobilon-P, Millipore) utilizing a semi-dry transfer apparatus (Bio-Rad) at 2 mA per 1 cm^2^ for 45 min (10% or 12% gel) or 15 V constant voltage for 30 min (13.5% gel with 6 M urea). Following transfer, the membranes were blocked with 2% skim milk in Tris-buffered saline containing 0.05% Tween 20 (TBST) for 30 min at room temperature (RT). Membranes were then incubated with primary anti-Atg8 (1:5000) [6], anti-Ape1 (1:10000) [16] anti-GFP (1:10000; JL-8, Clontech) antibodies for 60 min at RT. Subsequently, membranes were washed three times with TBST and treated with horseradish peroxidase (HRP)-labeled anti-rabbit or -mouse secondary antibodies (Promega) at a dilution of 1:5000 for 30 min, followed by an additional wash cycle in TBST. Chemiluminescent signals generated with the enhanced chemiluminescence (ECL) reagent (GE Healthcare) were detected on an IR-LAS 1000 imaging system (FUJIFILM).

### Fluorescence microscopy

Cells were cultured in SDCA medium to a density of about 5×10^7^ cells/ml, and autophagy was induced by addition of rapamycin. Cells were harvested, spun at RT with a microcentrifuge, and subjected to fluorescence microscopy using an IX83 inverted system microscope (Olympus) equipped with a UPlanSApo100×/1.40 Oil (Olympus) and a CoolSNAP HQ CCD camera (Nippon Roper). A U-FGFP and U-FMCHE filter sets (Olympus) were used for GFP/mNeonGreen and octadecyl rhodamine B (R18, Invitrogen)/FM4-64 staining visualization, respectively. Images were acquired using the MetaVue imaging software (Molecular Devices). For determination of IM length, fluorescence intensities of mNeonGreen-labeled Atg proteins were measured using the ‘linescan’ function of the MetaView software, and the full width at half maximum was calculated manually.

For R18 staining, cells were stained with 10 μg/ml of R18 (1 mg/ml stock dissolved in dimethyl sulfoxide) for 10 min in nutrient-rich medium at 30°C. After cells were washed three times with fresh medium, rapamycin was added to induce autophagy. FM 4–64 staining was performed as previously described [16].

## Results

### Localization of Atg8 to autophagy-related structures requires Atg8 cleavage but not Atg8-PE delipidation

**Fig 1:**
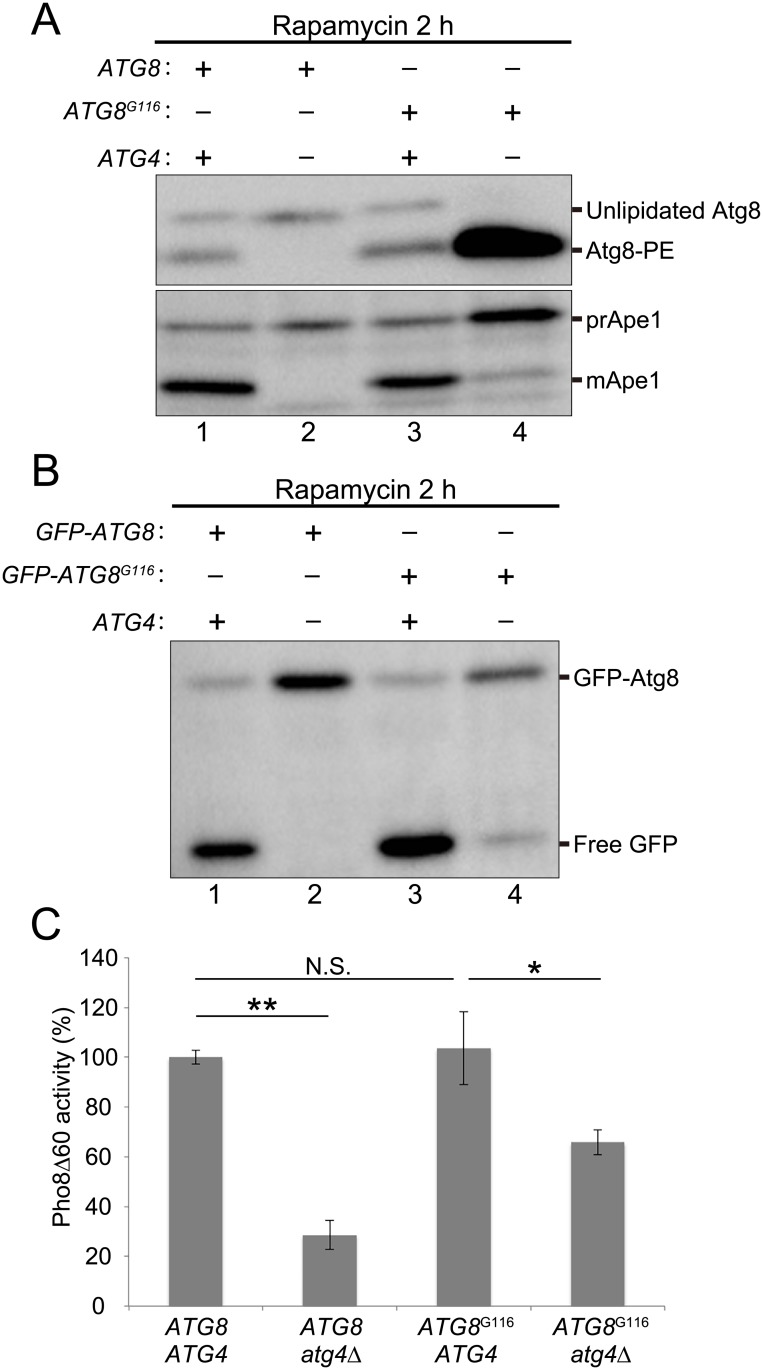
Autophagic activity of *ATG8*^G116^ *atg4*Δ cells. (A) *ATG8 atg4*Δ (KVY13) or *ATG8*^G116^ *atg4*Δ (GYS608) cells carrying an empty or Atg4-expressing plasmid were grown in SDCA medium to mid-log phase and treated with rapamycin for 2 h. Western blot analysis was performed using anti-Atg8 and anti-Ape1 antisera. Slower-migrating bands in the upper panel correspond to Atg8/Atg8^G116^ (Unlipidated Atg8), and faster-migrating bands correspond to Atg8-PE. Slower-migrating bands in the lower panel correspond to the precursor form of Ape1 (prApe1), and faster-migrating bands correspond to the mature Ape1 (mApe1). (B) *ATG8 atg4*Δ (KVY13) or *ATG8*^G116^ *atg4*Δ (GYS608) cells carrying the indicated plasmids were grown in SDCA medium to mid-log phase and treated with rapamycin for 2 h. Western blot analysis was performed with anti-GFP antibodies. Slower-migrating bands represent GFP-tagged Atg8 (GFP-Atg8), and faster-migrating bands represent cleaved GFP. (C) *ATG8*^G116^ (YOC5308) and *ATG8*^G116^ *atg4*Δ (YOC5309) cells were grown in SDCA medium to mid-log phase and shifted to SD(-N) medium and incubated for 4 h at 30°C. Then autophagic activity was measured by the alkaline phosphatase assay. Error bars indicate standard deviations. N.S. indicates not significant. *P < 0.05, **P < 0.01 (two-tailed Student’s *t*-test) (n = 3).

Cleavage of Atg8 by Atg4 is a prerequisite for Atg8-PE formation, which is in turn essential for localization of Atg8 to autophagy-related structures; thus, autophagosome formation is abolished in *atg4*Δ cells [8, 17]. When Atg8 lacking the C-terminal arginine residue (Atg8^G116^) is expressed in *atg4*Δ cells, cleavage of Atg8 can be bypassed; Atg8-PE is produced in the absence of Atg4, but autophagosome formation is still defective in this mutant [5]. We examined the autophagic activity of Atg8^G116^ *atg4*Δ cells by performing Ape1 maturation, GFP-Atg8 cleavage, and alkaline phosphatase assays. The results revealed that these cells are defective in autophagy (Fig 1).

**Fig 2:**
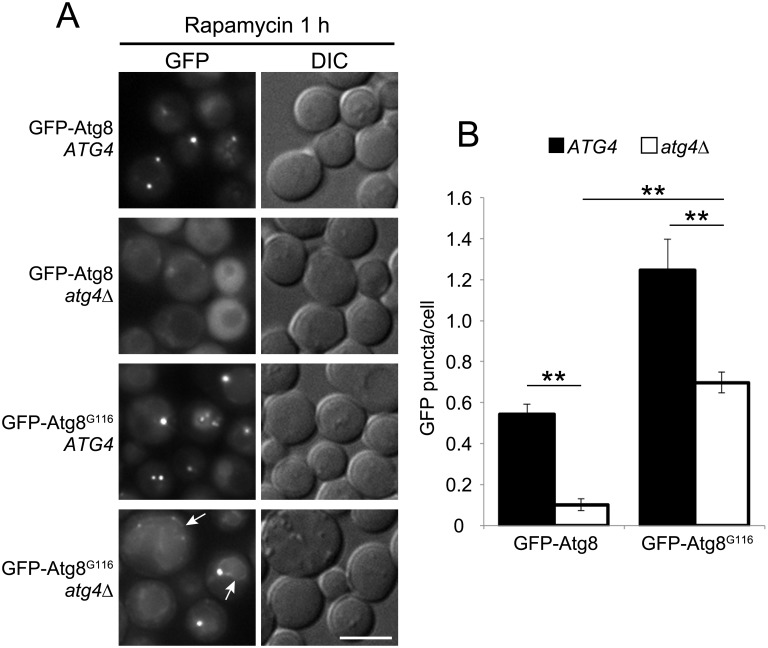
Localization of GFP-Atg8 and GFP-Atg8^G116^ in *atg4*Δ cells. (A) *GFP-ATG8 atg4*Δ (ORY0804) or *GFP-ATG8*^G116^ *atg4*Δ (GYS622) cells carrying an empty or Atg4-expressing plasmid were grown to mid-log phase in SDCA medium and treated with rapamycin for 1 h. Arrows indicate the vacuole rim. DIC, differential interference contrast. Scale bar: 5 μm. (B) Number of GFP-Atg8 puncta per cell. Error bars indicate standard deviations. **P < 0.01 (two-tailed Student’s *t*-test). At least 40 cells were counted for each experiment (n = 4).

It remains controversial whether delipidation of Atg8-PE is required for targeting of Atg8 to autophagy-related structures [5, 10, 11]. To address this issue, we first examined localization of Atg8 and Atg8^G116^ by fluorescence microscopy. In GFP-Atg8 expressing wild-type cells, GFP-Atg8 was visualized as puncta close to the vacuole, which corresponded to autophagy-related structures (Fig 2A). By contrast, the abundance of puncta was markedly reduced in *atg4*Δ cells (Fig 2A); this observation was supported by counting the number of puncta per cell (Fig 2B). This result is reasonable because lipidation of GFP-Atg8 is inhibited in the absence of Atg4. Next, we examined the localization of GFP-Atg8^G116^. GFP-Atg8^G116^ was detected as puncta in wild-type and *atg4*Δ cells (Fig 2A). Quantification of the puncta revealed that the number of puncta was significantly smaller in *atg4*Δ cells than in wild-type cells (Fig 2B). This decrease is probably caused by lacking the localization of GFP-Atg8^G116^ to at least autophagosomes in *atg4*Δ cells because autophagosome formation is severely defective in Atg8^G116^ *atg4*Δ cells [5]. Moreover, GFP-Atg8^G116^ exhibited a vacuolar membrane pattern in *atg4*Δ cells, as previously reported (Fig 2A, arrows) [5].

**Fig 3:**
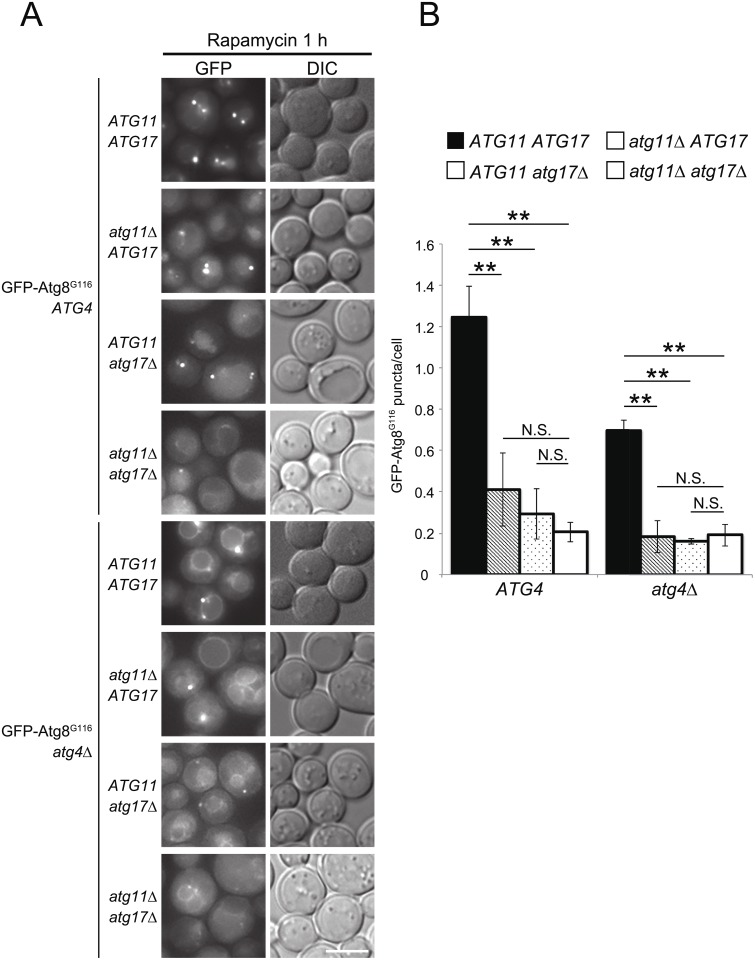
Localization of GFP-Atg8^G116^ in *atg4*Δ cells lacking the scaffold complex for autophagy-related structures. (A) *atg4*Δ (GYS622), *atg4*Δ *atg11*Δ (YOC5270), *atg4*Δ *atg17*Δ (YOC5269), and *atg4*Δ *atg11*Δ *atg17*Δ (YOC5271) cells expressing GFP-Atg8^G116^ and carrying an empty or Atg4-expressing plasmid were grown to mid-log phase in SDCA medium and treated with rapamycin for 1 h. Scale bar: 5 μm. (B) Number of GFP-Atg8^G116^ puncta per cell. Error bars indicate standard deviations. N.S., not significant. **P < 0.01 (two-tailed Student’s *t*-test). At least 40 cells were counted for each experiment (n = 4).

Autophagy-related structures disappear in *atg11*Δ *atg17*Δ cells because Atg11 and Atg17 are scaffold proteins playing a role in recruitment of Atg proteins [17, 18]. To determine whether the puncta observed in GFP-Atg8^G116^–expressing cells represented autophagy-related structures, we disrupted *ATG11* and *ATG17* genes in these strains. In *ATG4* cells expressing GFP-Atg8^G116^, the number of puncta decreased in *atg11*Δ, *atg17*Δ, and *atg11*Δ *atg17*Δ cells (Fig 3). In *atg4*Δ cells expressing GFP-Atg8^G116^, the number of puncta decreased upon disruption of *ATG11*, *ATG17*, or both (Fig 3A). Quantification revealed that the number of the puncta was at the basal level in *atg11*Δ, *atg17*Δ, and *atg11*Δ *atg17*Δ cells (Fig 3B). Therefore, the puncta observed in GFP-Atg8^G116^–expressing *ATG4* and *atg4*Δ cells are unlikely to be dead-end structures, and instead correspond to autophagy-related structures.

### Atg8-PE delipidation is important for IM expansion

The number of GFP-Atg8^G116^ dots observed in *ATG4* cells markedly decreased in *atg4*Δ cells (Fig 2), and these dots corresponded to autophagy-related structures (Fig 3). Together, these observations support the fact that the autophagosome is hardly generated in Atg8^G116^ *atg4*Δ cells. Next, we examined whether the IM expands in Atg8^G116^ *atg4*Δ cells by the prApe1-overexpression system, which enables visualization of the IM as a cup-shaped structure [2]. In this experiment, Atg8 and Atg8^G116^ were visualized by fusing green fluorescent mNeonGreen to their N-termini.

**Fig 4:**
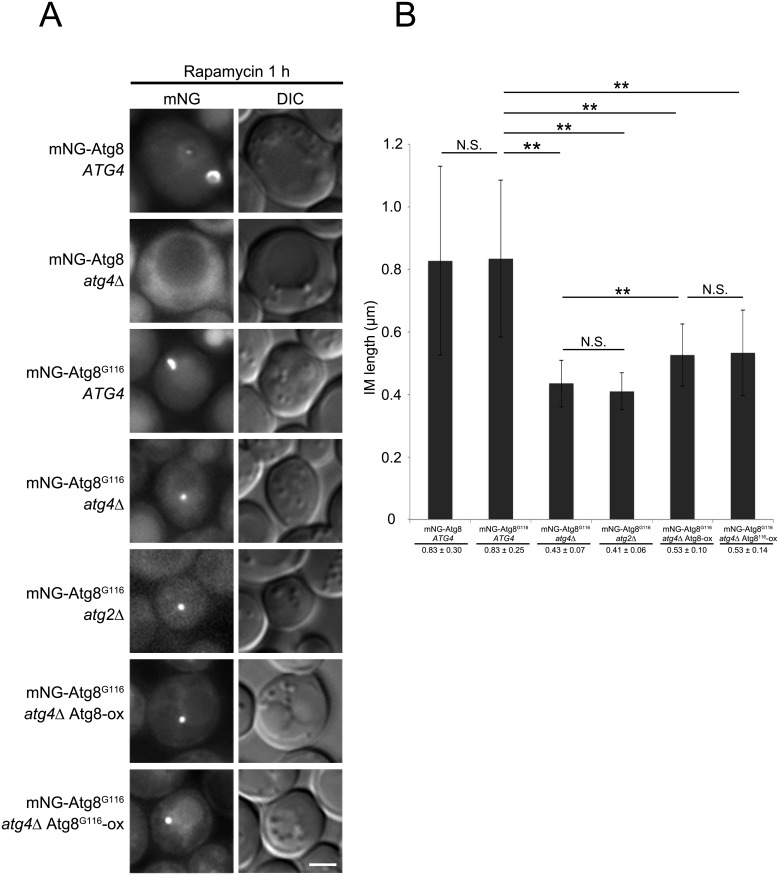
IM expansion in delipidation-mutant cells. (A) mNeonGreen(mNG)-Atg8 *atg4*Δ (YOC5272) and mNG-Atg8^G116^ *atg4*Δ (YOC5330) cells carrying an empty or Atg4-expressing plasmid, mNG-Atg8^G116^ *atg4*Δ *atg2*Δ (YOC5331) cells carrying an Atg4-expressing plasmid, and mNG-Atg8^G116^ *atg4*Δ (YOC5330) cells carrying an Atg8- or Atg8^G116^-overexpressing (ox) plasmid were grown to mid-log phase in SDCA medium containing CuSO_4_, and then treated with rapamycin for 1 h. Scale bar: 2 μm. (B) Lengths of IMs were measured. Error bars indicate standard deviations. **P < 0.01 (two-tailed Student’s *t*-test).

Cup-shaped IMs were visualized in wild-type cells expressing mNeonGreen-Atg8, but no autophagy-related structures were observed in *atg4*Δ cells due to their defect in cleavage (Fig 4A). mNeonGreen-Atg8^G116^ was visualized as a cup-shaped structure in wild-type cells, but as dots in *atg4*Δ and *atg2*Δ cells (Fig 4A). We observed no significant difference in IM lengths between wild-type cells expressing mNeonGreen-Atg8 and those expressing mNeonGreen-Atg8^G116^ (Fig 4B), indicating that cleavage by Atg4 does not have a large impact on IM lengths. The average IM lengths in mNeonGreen-Atg8^G116^ expressing wild-type, *atg4*Δ, and *atg2*Δ cells were 0.83 μm, 0.43 μm, and 0.41 μm, respectively (Fig 4B), indicating that the IM lengths in *atg4*Δ cells were significantly shorter than those in wild-type cells. In *atg2*Δ cells, Atg8^G116^ can localize to the VICS, but the IM does not expand [2, 17]. We detected no significant differences in IM lengths between Atg8^G116^ expressing *atg4*Δ and *atg2*Δ cells (Fig 4B). Thus, IM expansion was reduced to the basal level without delipidation.

**Fig 5:**
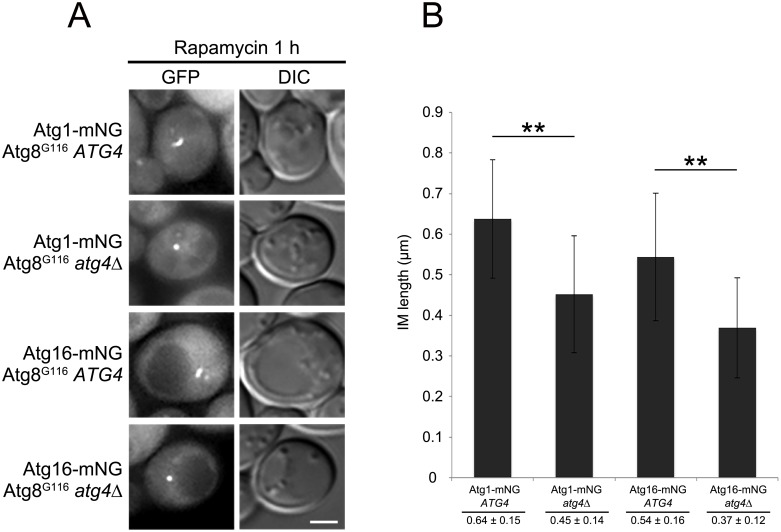
Lengths of Atg1 or Atg16-labeled IMs in delipidation-mutant cells. (A) Atg1-mNG Atg8^G116^ *atg4*Δ (YOC5469) and Atg16-mNG Atg8^G116^ *atg4*Δ (YOC5470) cells carrying an empty or Atg4-expressing plasmid were grown to mid-log phase in SDCA medium containing CuSO_4_, and then treated with rapamycin for 1 h. Scale bar: 2 μm. (B) Lengths of IMs were measured. Error bars indicate standard deviations. **P < 0.01 (two-tailed Student’s *t*-test).

In mammalian cells, the IM can expand without lipidation of Atg8 or Atg8 itself [19–21]. Therefore, we examined the IMs labeled with other marker proteins in Atg8^G116^ *ATG4* and Atg8^G116^ *atg4*Δ cells. Previously, our group have shown that Atg1 and Atg16 are localized to the IM [2]. Therefore, we examined localization of Atg1 and Atg16 in Atg8^G116^ *ATG4* or Atg8^G116^ *atg4*Δ cells overexpressing prApe1. In Atg8^G116^ *ATG4* cells, Atg1 and Atg16 were visualized as cup-shaped structures, whereas they are observed as dots in Atg8^G116^ *atg4*Δ cells (Fig 5A). The IM lengths of the structures in Atg8^G116^ *atg4*Δ cells were significantly shorter than those in Atg8^G116^ *ATG4* cells (Fig 5B). These results show that the IMs labeled with Atg1 and Atg16 cannot fully expand without delipidation. Taken together, we conclude that delipidation of Atg8-PE is important for IM expansion.

### Atg8-PE delipidation plays an important role in IM expansion other than supplying unlipidated Atg8

**Fig 6:**
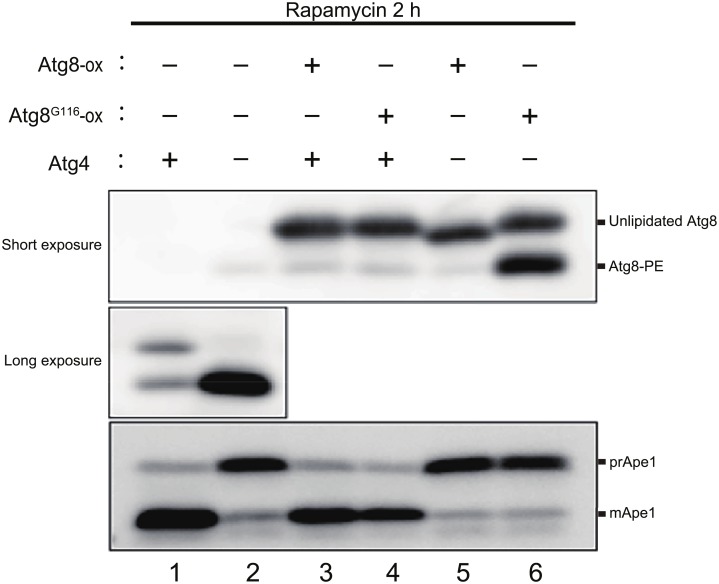
Autophagic activity of Atg8^G116^ *atg4*Δ cells overexpressing Atg8/Atg8^G116^. *ATG8*^G116^ *atg4*Δ (GYS608) cells expressing indicated proteins were grown in SDCA medium to mid-log phase, and then treated with rapamycin for 2 h. Western blot analysis was performed with anti-Atg8 and anti-Ape1 antisera. Short- and long-exposed images were shown. Slower-migrating bands in the upper and middle panels correspond to unlipidated Atg8, and faster-migrating bands correspond to Atg8-PE. Slower-migrating bands in the lower panel correspond to the precursor form of Ape1 (prApe1), and faster-migrating bands correspond to the mature Ape1 (mApe1). ox indicates overexpression.

Most Atg8^G116^ expressed in *atg4*Δ cells was detected as Atg8-PE (Fig 6, lane 2) [5]. We hypothesized that the shortage of unlipidated Atg8^G116^ caused a defect in IM expansion in Atg8^G116^-expressing *atg4*Δ cells as shown in Fig 4. To address this issue, we overexpressed Atg8 or Atg8^G116^ in Atg8^G116^ background cells. In Atg8^G116^ *ATG4* cells, overexpressed Atg8 and Atg8^G116^ were mostly detected as an unlipidated form, and Atg8-PE was detected as minor bands (Fig 6, lanes 3 and 4). Overexpression of Atg8 and Atg8^G116^ in Atg8^G116^ *ATG4* cells did not affect the maturation of Ape1 (Fig 6, lanes 3 and 4). When Atg8 was overexpressed in Atg8^G116^ *atg4*Δ cells, most Atg8 was detected as an unlipidated form (Fig 6, lane 5). On the other hand, Atg8-PE as well as unlipidated Atg8 was detected in Atg8^G116^ *atg4*Δ cells overexpressing Atg8^G116^ (Fig 6, lane 6). Nevertheless, the maturation of Ape1 was severely defective in Atg8^G116^ *atg4*Δ cells overexpressing Atg8^G116^ (Fig 6, lane 6). These results indicate that only the presence of unlipidated Atg8^G116^ in addition to Atg8-PE is insufficient to recover autophagic activity in Atg8^G116^ *atg4*Δ cells, suggesting that the shortage of unlipidated Atg8^G116^ is not the main cause of a defect in autophagy in these cells.

Next, we measured IM lengths in Atg8^G116^ *atg4*Δ cells overexpressing Atg8 or Atg8^G116^. The IM lengths in both strains were significantly longer than those in Atg8^G116^ *atg4*Δ cells but significantly shorter than those in Atg8^G116^ *ATG4* cells (Fig 4). This result suggests that the defect in IM expansion in Atg8^G116^ *atg4*Δ cells cannot be fully recovered by supplying unlipidated Atg8^G116^. Taken together, we conclude that delipidation of Atg8 itself plays an important role in efficient IM expansion.

### Autophagic membranes are present in cells defective in Atg8-PE delipidation, but not in *atg2*Δ cells

**Fig 7:**
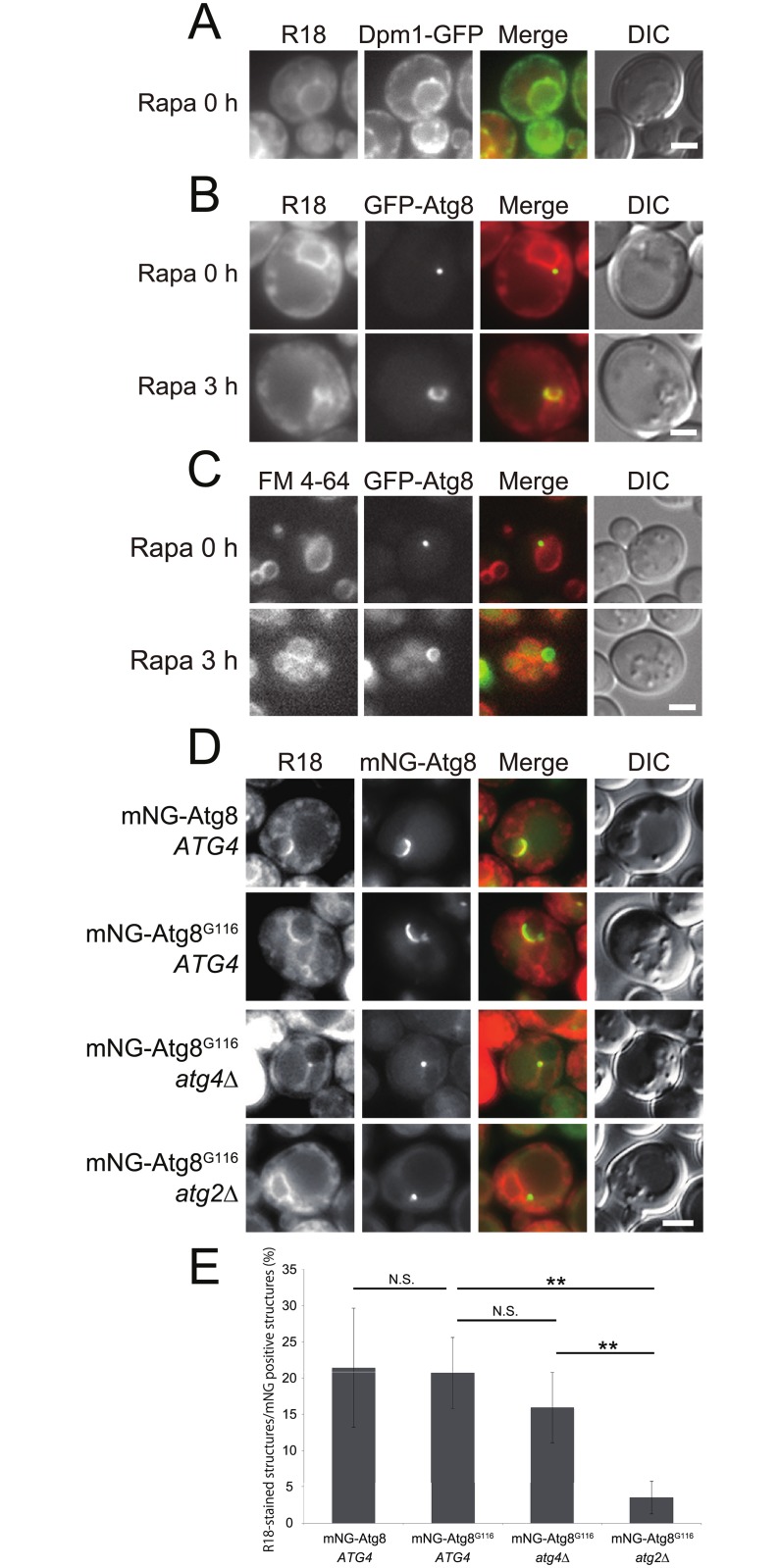
Staining of autophagy-related structures with octadecyl rhodamine B. (A) Dpm1-GFP expressing cells (YCK445) harboring the pYEX-BX[prApe1] plasmid were grown in SDCA medium containing CuSO_4_ and stained with octadecyl rhodamine B (R18). After the cells were washed with fresh medium, they were observed by fluorescence microscopy. (B) Wild-type cells harboring pRS314[GFP-Atg8] and pYEX-BX[prApe1] plasmids were grown in SDCA medium containing CuSO_4_ and stained with R18. After the cells were washed with fresh medium, they were treated with rapamycin for 3 h. (C) Wild-type cells harboring pRS314[GFP-Atg8] and pYEX-BX[prApe1] plasmids were grown in SDCA medium containing CuSO_4_ and stained with FM 4–64. After the cells were incubated with fresh medium without dye for 30 min, they were treated with rapamycin for 3 h. (D) mNG-Atg8 *atg4*Δ (YOC5272) and mNG-Atg8^G116^ *atg4*Δ (YOC5330) cells carrying an empty or Atg4-expressing plasmid and mNG-Atg8^G116^ *atg4*Δ *atg2*Δ (YOC5331) cells carrying an Atg4-expressing plasmid were grown to mid-log phase in SDCA medium containing CuSO_4_, and then stained with R18. After the cells were washed with fresh medium, they were treated with rapamycin for 1 h. Scale bar: 2 μm. (E) Frequencies of mNG-positive structures stained with R18. Error bars indicate standard deviations. N.S., not significant. **P < 0.01 (two-tailed Student’s *t*-test). At least 40 cells were counted for each experiment (n = 4).

We tested several kinds of lipophilic fluorescent dyes and found that octadecyl rhodamine B (R18) preferentially stained the ER labeled with Dpm1-GFP, an ER transmembrane marker, with slight staining of the vacuolar membrane, under nutrient-rich conditions (Fig 7A). In R18-stained cells, GFP-Atg8 was visualized as a dot close to the vacuole without rapamycin treatment (Fig 7B). A cup-shaped IM emerged after treatment with rapamycin, and the IM was labeled with R18 (Fig 7B), suggesting that lipids constituting the IM can be labeled by R18 staining. Because the vacuolar membrane was slightly stained with R18, we also explored the possibility that the IM is stained with FM 4–64, a lipophilic dye that labels the vacuolar membrane. The vacuolar membrane was stained with FM 4–64 and treated with rapamycin. In contrast to R18, the IM was not stained with FM 4–64 (Fig 7C). Therefore, R18 preferentially labels IM lipids, whereas FM 4–64 does not.

Cup-shaped IMs were stained with R18 in *ATG4* cells expressing mNeonGreen-Atg8 or mNeonGreen-Atg8^G116^ (Fig 7D). In mNeonGreen-Atg8^G116^ expressing *atg4*Δ cells, mNeonGreen-labeled structures were also stained with R18, and there were no significant differences in frequency of R18-labeling relative to wild-type cells (Fig 7D and 7E). On the other hand, mNeonGreen-labeled structures in *atg2*Δ cells barely stained with R18 (Fig 7D and 7E). These results suggest that mNeonGreen-Atg8^G116^ expressing *atg4*Δ cells are capable of recruiting lipids to the IM, whereas *atg2*Δ cells are not.

## Discussion

In this study, we demonstrated that delipidation of Atg8-PE by Atg4 is dispensable for targeting of Atg8-PE to the VICS, but required for expansion of the IM (Fig 4). We also obtained a result suggesting that biological membranes exist at the Atg8-labeled structures in delipidation-defective cells (Fig 7). From these facts, we think that Atg4 is involved in efficient expansion of the IM by cleaving Atg8-PE localized at the VICS.

Here we show that IM expansion is impaired in Atg8^G116^ *atg4*Δ cells at the same level as in Atg8^G116^ *atg2*Δ cells (Fig 4). On the other hand, previous studies showed that a small number of closed autophagosomes are formed in Atg8^G116^ *atg4*Δ cells, whereas no autophagosomes are detected in *atg2*Δ cells [5, 22, 23]. These facts suggest that the IM in Atg8^G116^ *atg4*Δ cells has an ability to become a closed autophagosome, whereas that of Atg8^G116^ *atg2*Δ cells does not. Thus, Atg8-PE delipidation by Atg4 is unlikely to play a role in closure of the IM; instead, the delipidation reaction is mainly involved in IM expansion. We also found that autophagy-related structures in Atg8^G116^ *atg4*Δ cells were stained with lipophilic dye R18, whereas those in Atg8^G116^ *atg2*Δ cells were not (Fig 7D and 7E). Based on these observations, we hypothesize that Atg2 is involved in recruitment of lipids to the VICS, and that subsequent delipidation of Atg8-PE by Atg4 serves to expand the IM. The minimal autophagic activity in Atg8^G116^ *atg4*Δ cells might be explained by the presence of lipids in the autophagy-related structures.

In *ATG4* cells, Atg8-PE is produced, and GFP-Atg8 localizes to autophagy-related structures (Figs 1A and 2A), suggesting that Atg8 localized to autophagy-related structures is conjugated to PE. On the other hand, when we visualized the IM, we demonstrated that the IM length of Atg8^G116^ *atg4*Δ cells did not significantly differ from that in Atg8^G116^ *atg2*Δ cells (Fig 4), indicating that Atg8-PE delipidation is required for IM expansion. Therefore, we assume that delipidation activity of Atg4 is initially repressed at the VICS, but derepressed upon IM expansion.

Previous reports proposed that delipidation supplies Atg8^G116^ to promote autophagosome formation [5, 11]. However, overexpression of Atg8^G116^ cannot rescue the defect in autophagic activity in *atg4*Δ *atg8*Δ cells [10, 11]. We examined the level of unlipidated Atg8^G116^ in Atg8^G116^-overexpressing Atg8^G116^ *atg4*Δ cells and detected a certain amount of unlipidated Atg8^G116^ (Fig 6). However, IM expansion and autophagic activity were not rescued in these cells (Figs 4 and 6), suggesting that the role of Atg8-PE delipidation is not limited to supplying unlipidated Atg8^G116^. Based on these results, we propose three possible scenarios: (1) a cycle of lipidation and delipidation is involved in IM expansion; (2) because accumulation of Atg8-PE at the VICS may disturb IM expansion, surplus Atg8-PE must be delipidated by Atg4; or (3) the site of delipidation occurs is important, i.e., Atg8-PE must be delipidated at a specific membrane, and then Atg8^G116^ plays a role at the site of delipidation. Notably, IM lengths slightly increased by overexpression of Atg8 or Atg8^G116^ in Atg8^G116^ *atg4*Δ cells (Fig 4), whereas autophagic activity remained unchanged (Fig 6, lanes 2, 5 and 6). These facts suggest that excess unlipidated Atg8/Atg8^G116^ affects the shape of the Atg8-labeled structures but does not improve the activity of autophagosome formation.

Lipidation of Atg8 mediates the tethering and hemifusion of liposomes *in vitro* [24]. Based on this fact, Atg8-PE is thought to be involved in IM expansion. Here, we report that delipidation of Atg8-PE is also essential for IM expansion, implying that Atg8^G116^ plays a role in this step. Addition of delipidated Atg8 to the *in vitro* Atg8-PE conjugation reaction may modulate the hemifusion activity of Atg8-PE.

The ER marker proteins GFP-HDEL and Dpm1-GFP do not label the IM [2], indicating that these proteins do not transit to the IM from the ER. However, we found that a lipophilic dye R18 labeled the IM (Fig 7B). This result could be interpreted in two ways. First, the dye could be transported to the IM via canonical vesicular trafficking pathways from the ER. Alternatively, lipids could be supplied from the ER to the IM directly. We prefer the latter ‘lipid flow’ model because we have never observed any vesicle-like structures around the IM, despite intensive observation by electron microscopy (data not shown). Future work should seek to clarify the mechanisms involved in transport of lipids from the ER to the IM. It is worth noting that we cannot exclude the possibility that other organelles supply lipids to the IM.

Further studies of Atg4 will be necessary to reveal the mechanisms of IM expansion mediated by delipidation of Atg8-PE.

## Abbreviations

Atg: autophagy-related
IM: isolation membrane
PE: phosphatidylethanolamine
